#  Effects of hip joint position and intra-capsular volume on hip joint intra-capsular pressure: a human cadaveric model

**DOI:** 10.1186/1749-799X-4-8

**Published:** 2009-04-02

**Authors:** Chi-Hung Yen, Hon-Bong Leung, Paul Yun-Tin Tse

**Affiliations:** 1 Department of Orthopaedics and Traumatology, Kwong Wah Hospital, 25 Waterloo Road, Yaumatei, The Hong Kong Special Administrative Region; 2 Department of Orthopaedics and Traumatology, Queen Mary Hospital, Pokfulam, The Hong Kong Special Administrative Region

## Abstract

**Background:**

Increase in hip intra-capsular pressure has been implicated in various hip pathologies, such as avascular necrosis complicating undisplaced femoral neck fracture. Our study aimed at documenting the relationship between intra-capsular volume and pressure in various hip positions.

**Methods:**

Fifty-two cadaveric hips were studied. An electronic pressure-monitoring catheter recorded the intra-capsular hip pressure after each instillation of 2 ml of normal saline and in six hip positions.

**Results:**

In neutral hip position, the control position for investigation, intra-capsular pressure remained unchanged when its content was below 10 ml. Thereafter, it increased exponentially. When the intra-capsular volume was 12 ml, full abduction produced a 2.1-fold increase (p = 0.028) of the intra-capsular hip joint pressure; full external rotation and full internal rotation increased the pressure by at least 4-fold (p < 0.001). Conversely, there was a 19% (p = 0.046) and 81% (p = 0.021) decrease in intra-capsular hip joint pressure with flexion of the hip joint to 90-degree and 45-degree, respectively.

**Conclusion:**

Intra-capsular pressure increases with its volume, but with a wide variation with different positions. It would be appropriate to recommend that hips with haemarthrosis or effusion should be positioned in 45-degree flexion.

## Introduction

Increased intra-capsular pressure of the hip joint secondary to effusion or haemarthosis was recorded in various hip pathologies, such as femoral neck fracture[1-4], transient synovitis[5-8], juvenile chronic arthritis[5], slipped proximal femoral epiphysis[9], and in contused hips[10]. The raised pressure results in not only pain but also limited range of motion. [1,9,11] Furthermore, the accompanied increase in the hip joint pressure is thought to be important in the pathogenesis of Legg-Perthes' disease[12], and the progression of aseptic loosening of total hip prosthesis[13]. Although it remains controversial, the accompanied bony venous congestion might partially account for the avascular necrosis and non-union after femoral neck fracture. [9,10,14-16]

Schwarz first documented the rise of intra-capsular pressure by instilling Ringer's solution in cadaveric hips. [17] However, his pioneered work failed in depicting the pressure-volume relationship. If one can predict the intra-capsular pressure by knowing its volume (e.g. by means of ultrasound or computer tomogram), it will be clinically important as high intra-capsular pressure might warrant aggressive intervention.

Stromquvist recognised the intra-capsular hip pressure would change with hip positions. [4] He suggested patients should be nursed with their hips in semi-flexion to lower the intra-capsular pressure. Nonetheless, since his finding was based on patients with femoral neck fracture, generalising the conclusion to other situations should be cautious.

The primary objective of this project was to estimate the hip pressure-volume relationship with refined methodology, improved quality of data analysis and reporting. The secondary objective was to investigate the effect of hip position on the intra-capsular pressure, when the joint was loaded with various amount of fluid.

## Materials and methods

Procurement process was initiated at the time of certified death. To guard against biohazard, only subjects without infectious disease (Category one bodies)[18] were recruited. All these bodies were assessed by the authors (CHY and HBL) to ensure *rigor mortis *had not set in, the lower limbs of these subjects had no clinically detectable abnormality, and the hip joints had unimpaired passive range of movement. For bodies that we identified to be eligible for recruitment, as an additional measure to exclude degeneration and avascular necrosis of the hip, we approached the families to enquire for any past complain over the hip joint by the diseased patients. If that was negative, informed written consent was obtained from the next of kin.

Handling of these bodies followed guidelines jointly proposed by the Department of Health, the Hospital Authority, and the Food and Environmental Hygiene Department of the Government of Hong Kong. [18] All procedures were performed inside a Biosafety Level-2 suite. Personal protection equipment such as face shields, protective gloves and impermeable gowns were utilised according to the recommendation made by the Center for Disease Control and Prevention on Biosafety in Microbiological and Biomedical Laboratories. [19]

**Figure 1:**
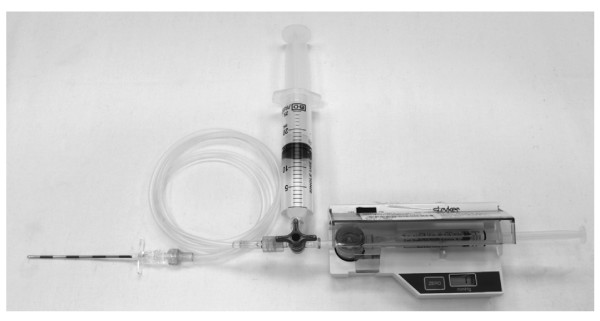
**The digital pressure monitor system used to measure intra-capsular pressure of cadaveric hip**. The whole system consists of a 18-gauge 3.25-inch epidural needle (Perican^® ^with Tuohy bevel, B. Braun Melsungen AG, Brazil), an unyielded 54-inch connection tubing designed for arterial line measurement, a 3-way stopcock, a 25 ml syringe, and the Quick Pressure Monitor Set mounted inside the Stryker Pressure Monitor (Stryker Inc, Cedex, France). The Quick Pressure Monitor Set consists of a membrane drum, and a 3 ml syringe. It is manufactured by the same company as the consumable for the pressure monitor. The system was primed with saline.

The cadavers were positioned supine. The hip joints were approached via open anterior approach. Dissection was carried out lateral to the femoral neurovascular bundle. Muscles anterior to the hip joint were retracted to expose the joint capsule. The pressure monitor system was assembled as shown in Figure 1. It consisted of a digital pressure monitor set (REF 295-1) manufactured by Stryker^® ^Instruments, Michigan USA This monitor set could be self-calibrated and the resolution of measurement was 1 mmHg over the range of -10 to 200 mmHg. When the tip of the 18-gauge epidural needle was positioned on the hip joint capsule, just before puncturing the capsule, the monitor set was primed with saline and calibration was then performed.

Under direct visualisation, the hip joint capsule was punctured by the epidural needle. Muscle retraction was then released before the entry pressure was recorded. The joint was then aspirated dry with the volume of aspiration documented. Normal saline was instilled in 2 ml increments and the intra-capsular pressure was recorded in six standardised hip positions in the following sequence. The neutral position was obtained when the cadaver was placed supine on a hard surface with his knees and heels rest on the surface and his feet brought together to standardise the degree of rotation. Additional positions included 45-degree flexion, 90-degree flexion, full internal rotation in zero-degree hip extension, full external rotation in zero-degree hip extension, and lastly, full abduction in zero-degree hip extension. After each instillation of saline or change in hip position, we gently rocked the hip for ten seconds so as to attain equilibrium of the intra-capsular pressure before pressure readings were taken.

End point was reached after attaining the maximal intra-capsular pressure in neutral hip position measurable by the pressure monitor system, being 200 mmHg. After taking the hip through the six defined positions, no further volume of fluid would be instilled.

Hips with haemarthrosis, turbid joint fluid and effusion (if aspiration yielded more than 10 ml) were excluded. Those hips with capsular tear, more than a single trial of joint puncture, or dislodged epidural needle, were also excluded in view of possible leakage. The procedure was adjourned when there was a pool of fluid at the puncture site or a drop in pressure regardless of increasing volume of saline injected. These findings suggested leakage. All data harvested from these hip joints with leakage were discarded from analysis, as we could not be certain when the leakage started to occur.

The whole procedure should finish before the onset of *rigor mortis*, an extra-articular constraint. It usually started around six hours after death.

Statistical analysis was performed by SigmaStat for Windows version 3.0 (SPSS Inc, Chicago, USA). Two-way repeated measures ANOVA (Holm-Sidak pairwise comparison test) was used to assess the interaction between hip position and intra-capsular volume. Overall significant level was set at 0.05.

## Results

Thirty-two Chinese cadavers, with 64 hip joints, were recruited in the study. Their age ranged from 66 to 97 years (mean = 84.2, standard deviation = 6.7). Twenty-two cadavers (69.8%) were female. Their body height varied from 124 to 168 cm (mean = 144.8, standard deviation = 7.3) whereas body weight varied from 41 to 62 kg (mean = 54.8, standard deviation = 4.5). The body mass index ranged from 16.1 to 25.2 kgm^-2 ^(mean = 21.1, standard deviation = 2.0).

Twelve hip joints were excluded from the study because the epidural needles were dislodged upon releasing the muscle retraction (8 cases), presence of a pool of saline (1 case) or a decrease in intra-capsular pressure despite instilling further volume of saline (3 cases). Among all the studied hips, no macroscopic capsular tear was noted. No hips had haemarthrosis or turbid joint fluid.

The entry pressure varied from -2 to 2 mmHg. All hip joints yielded dry aspiration.

**Figure 2:**
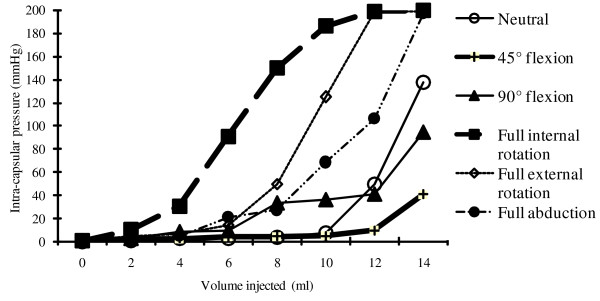
**Relationship between intracapsular hip joint pressure and hip joint volume according to six standardised hip joint positions**.

In neutral position, the hip joint pressure started to increase exponentially when its content exceeded 10 ml (Figure 2). When the volume of intra-articular fluid exceeded 4 ml, 45-degree hip flexion consistently showed the lowest pressure (p < 0.001) and full internal rotation in zero-degree extension yielded highest pressure (p < 0.001).

When the intra-capsular volume was 12 ml, compared to the neutral position, full abduction produced a 2.1-fold increase (p = 0.028) of intra-capsular hip joint pressure; full external rotation and full internal rotation increased the pressure by at least 4-fold (p < 0.001) (beyond measurable limit of the system) (Table 1). Conversely, there was a 19% (p = 0.046) and 81% (p = 0.021) decrease in intra-capsular hip joint pressure with flexion of the hip joint to 90-degree and 45-degree, respectively. At 14 ml intra-capsular volume, the intra-capsular pressure increased significantly. Even the hip joint was flexed to 45-degree, the mean intra-capsular volume remained higher than 40 mmHg, a level considered as dangerously high. [2]

**Table 1:** Intracapsular hip joint pressure according to hip joint volume and hip joint position.

	**Intracapsular hip joint volume**								
	0 ml	2 ml	4 ml	6 ml	8 ml	10 ml	12 ml	14 ml	p-value of ANOVA for different joint volume

**Hip joint position**									

Neutral	0.0 (0.0)	1.2 (0.6)	2.3 (2.1)	3.1 (0.6)	4.1 (1.7)	7.9 (5.5)	49.8 (15.9)	137.6 (30.9)	0.00

45° flexion	1.1 (1.5)	2.7 (1.5)	2.3 (2.1)	3.7 (1.5)	4.4 (1.9)	4.7 (2.1)	9.5 (4.8)	40.7 (16.1)	0.00

90° flexion	0.9 (1.3)	2.8 (1.8)	8.7 (7.2)	9.4 (7.0)	33.0 (16.9)	36.0 (15.5)	40.6 (16.3)	94.1 (25.1)	0.00

Full internal rotation	0.8 (1.3)	10.1 (4.8)	30.5 (42.8)	91.1 (21.5)	149.5 (34.0)	185.9 (30.5)	198.6 (10.3)	199.1 (6.7)	0.00

Full external rotation	0.8 (1.2)	4.2 (1.9)	6.2 (7.8)	14.1 (5.6)	50.0 (24.2)	124.8 (16.5)	198.2 (13.2)	198.5 (10.7)	0.00

Full abduction	0.2 (0.9)	1.9 (1.9)	4.8 (6.5)	21.1 (11.8)	27.4 (14.6)	68.5 (19.5)	105.8 (26.4)	196.9 (7.3)	0.00

p-value of ANOVA for different hip positions	0.99	0.12	0.04	0.00	0.00	0.00	0.00	0.00	

Values and blankets represent the mean pressure and standard deviation respectively. Units are in mmHg.

## Discussion

Schwarz [17] was honoured for pioneering hip pressure-volume relationship delineation. Our cadaveric model was based on his methodology but with a number of modifications. For example, open anterior approach was utilised as it was more direct, thus decreasing the chance of needle dislodgement. This approach also allowed visualisation of leakage if present. Instillation in 2 ml increment could better delineate the pressure-volume relationship. We rocked the hip after each instillation of fluid to equilibrate the effective hip joint cavity. Without practising such maneuver in our pilot study, the instantaneous intra-capsular pressure was quite high. Increased sample size could narrow the confidence interval. Pressure was measured by a commercial pressure transducer, which had been validated to have high accuracy. [20]

Regrettably, due to logistic reasons, the hips could not be examined radiologically to rule out intra-articular pathologies which could significantly affect the result. We were also unable to monitor the pressure through the range of motion and in their combination (e.g. 45-degree flexion with full internal rotation and abduction). Moreover, this study suffered from a few drawbacks that limited generalisation of data to *in vivo *circumstances. Firstly, in a cadaver model, normal soft tissue tension generated by muscle tone could not be restored. Secondly, we could not guard against minute leakage and uneven distribution of fluid inside the hip joint. Thirdly, weight of the leg and the external force applied by the authors in maintaining the defined hip position were not standardised. And since all subjects were elderly Chinese, extrapolating the information to younger age group and other ethnicities should be cautious. Finally, we only elected to test six positions in either coronal or sagittal plane. But the position that yielded the highest and lowest pressure might locate somewhere between the two planes, and with the degree of rotation not tested in our study.

We would like to alert readers on interpreting the sigmoid curve volume-pressure relationship when the hip was positioned in full internal rotation. This finding could be erroneous as the plateau effect was due to our limitation in recording pressure higher than 200 mmHg. Furthermore, leakage might start to occur when the pressure increased to this high level. Having said that, knowing the exact pressure might not be of clinical importance as 200 mmHg is already well above the critical perfusion pressure. [2,14] Drake et al reported a pressure of 40 mmHg could already jeopardise the femoral head perfusion. [2]

In neutral hip position, the intra-capsular pressure approached that critical perfusion pressure when 12 ml of 0.9% saline was instilled into the joint (Figure 2). However, in certain hip joint positions, such dangerously high pressure was attained at a much lower intra-capsular volume. Only 6 ml, 8 ml and 10 ml was required to exceed the critical intra-capsular pressure in full internal rotation, full external rotation, and full abduction respectively. The hip capsule, being reinforced by multiple ligamentous condensations, is not elastic under physiological condition. [21-23] Internal rotation of hip particularly tighten the ischiofemoral ligament and lateral arm of the iliofemoral ligament. The iliofemoral ligament of Bigelow is taut on external rotation. [24] The ischiofemoral ligament checkreins abduction. [25] Flexion, however, places least tension on these non-yielding ligaments. [24]

Although the actual volume of effusion or haemarthrosis was never known in clinical setting,[26] our finding suggested a simple measure could avoid undue intra-capsular pressure by paying respect to the hip position. Based on our data on intra-capsular pressure with reference to the hip positions, care should be exercised to avoid skin or skeletal traction of the affected hip in full external or internal rotation. Provided the intra-capsular volume is less than 12 ml, positioning the hip in 45-degree of flexion can confer a safe intra-capsular pressure below 40 mmHg. Clinically, this position can be simply accomplished by resting the affected leg on two pillows or a Thomas splint with Pearson knee attachment. For hip joint with its content estimated to be more than 14 ml, no hip position was found to be able to attain pressure lower than the critical perfusion level. The only way to maintain a safe level of intra-capsule pressure will be continuous aspiration or open drainage.

Although femoral neck fracture was not investigated in our study, we expect the above discussion might not be valid for displaced fracture. Drake et al reported that the volume of blood that could be aspirated from hip with displaced femoral neck fracture never exceeded 5 cc. [2] Crawfurd also documented that the intra-capsular pressure was higher in Garden [27] Grade I and II than in Garden Grade III and IV with an average of 66.4 mmHg and 28 mmHg respectively. [1] Although no concrete explanation was made, it might be in the event of displaced femoral neck fracture, not only the capsule was torn but also the intra-medullary cavity was rendered communicating with the joint proper. The intra-medullary venous system can effectively drain the hemarthrosis. Before further work is done, projecting our finding into this clinical scenario should be cautious.

From our clinical observation, patients with hip disorders were also commonly noted to rest their hips in flexion. No scientific explanation had been made to account for this apart from empirically relating it to preferential spasm of hip flexors. In our study, we demonstrated that in this particular position of hip, the joint conferred the lowest possible pressure. Relaxing the capsular ligaments in hip flexion could be the reason. An *in vivo *study might provide a better insight in this issue.

## Conclusion

In neutral hip position, joint pressure remained low until its content exceeded 10 ml. Afterwards, its pressure rose exponentially. Position of the hip joint affected the intra-capsular pressure. For the specific positions being tested, full internal rotation resulted in highest pressure, followed by full external rotation and full abduction. Forty-five-degree hip flexion yielded lowest pressure. We recommended to position hips with undisplaced femoral neck fracture or with effusion in 45-degree flexion to ensure low intra-capsular pressure.

## Competing interests

The authors declare that they have no competing interests.

## Authors' contributions

All authors had substantial contributions to conception and design, analysis and interpretation of data, drafting and giving final approval to the manuscript. CHY and HBL were responsible to the acquisition of data.
